# TNFα Transport Induced by Dynamic Loading Alters Biomechanics of Intact Intervertebral Discs

**DOI:** 10.1371/journal.pone.0118358

**Published:** 2015-03-03

**Authors:** Benjamin A. Walter, Morakot Likhitpanichkul, Svenja Illien-Junger, Peter J. Roughley, Andrew C. Hecht, James C. Iatridis

**Affiliations:** 1 Leni & Peter W. May Department of Orthopaedics at the Icahn School of Medicine at Mount Sinai, New York, NY, United States of America; 2 Department of Biomedical Engineering, The City College of New York, New York, NY, United States of America; 3 Shriners Hospital for Children, Montreal QC, Canada; Leibniz Institute for Age Research—Fritz Lipmann Institute (FLI), GERMANY

## Abstract

**Objective:**

Intervertebral disc (IVD) degeneration is an important contributor to the development of back pain, and a key factor relating pain and degeneration are the presence of pro-inflammatory cytokines and IVD motion. There is surprisingly limited understanding of how mechanics and inflammation interact in the IVD. This study investigated interactions between mechanical loading and pro-inflammatory cytokines in a large animal organ culture model to address fundamental questions regarding (i.) how inflammatory mediators arise within the IVD, (ii.) how long inflammatory mediators persist, and (iii.) how inflammatory mediators influence IVD biomechanics.

**Methods:**

Bovine caudal IVDs were cultured for 6 or 20-days under static &amp; dynamic loading with or without exogenous TNFα in the culture medium, simulating a consequence of inflammation of the surrounding spinal tissues. TNFα transport within the IVD was assessed via immunohistochemistry. Changes in IVD structural integrity (dimensions, histology &amp; aggrecan degradation), biomechanical behavior (Creep, Recovery &amp; Dynamic stiffness) and pro-inflammatory cytokines in the culture medium (ELISA) were assessed.

**Results:**

TNFα was able to penetrate intact IVDs when subjected to dynamic loading but not static loading. Once transported within the IVD, pro-inflammatory mediators persisted for 4–8 days after TNFα removal. TNFα exposure induced changes in IVD biomechanics (reduced diurnal displacements &amp; increased dynamic stiffness).

**Discussion:**

This study demonstrated that exposure to TNFα, as might occur from injured surrounding tissues, can penetrate healthy intact IVDs, induce expression of additional pro-inflammatory cytokines and alter IVD mechanical behavior. We conclude that exposure to pro-inflammatory cytokine may be an initiating event in the progression of IVD degeneration in addition to being a consequence of disease.

## Introduction

Inflammation is emerging as an important contributor to the pathogenesis of painful intervertebral disc (IVD) degeneration [1, 2], however, the specific role it plays in disease progression remains unclear. Pro-inflammatory cytokines can induce cellular changes that are characteristic of degeneration [3–8] and the expression of pro-inflammatory cytokines is correlated with aging and the severity of IVD degeneration [9–11]. It remains unclear how pro-inflammatory cytokines arise during disease and whether their presence is a contributor to, or consequence of, the disease process. The overall goal of this study was to investigate the fundamental questions regarding how inflammatory mediators arise within the IVD, how long inflammatory mediators persist, and how inflammatory mediators influence IVD biomechanics.

Injury and/or inflammation of spinal structures surrounding the IVD (i.e. spinal ligaments, vertebrae, and facet joints) are associated with spinal pathology [12–16] yet it remains unknown if inflammatory mediators, possibly resulting from inflamed spinal tissues, can penetrate intact IVDs. The acute response to tissue injury involves the expression of multiple pro-inflammatory cytokines including TNFα, IL-1β and IL-6 [17, 18]. This local increase in the concentration of inflammatory mediators immediately surrounding the IVD may provide another source of elevated inflammatory mediators within the IVD, as the concentration gradient would favor transport into the IVD. However, it is not known whether pro-inflammatory cytokines outside the IVD can penetrate a healthy IVD, which is considered ‘immune-privileged’ due to its lack of vasculature and slow transport kinetics.

Mechanical factors are also known to contribute to the progression of IVD degeneration [19] and may interact with the inflammatory component of the disease through enhancing transport of pro-inflammatory cytokines. The dominant mode of transportation for pro-inflammatory cytokines within the IVD remains unclear, however dynamic mechanical loading plays an important role in enhancing molecular transport of large solutes within cartilaginous tissues, through the addition of convective fluid flow [20, 21]. Solute size is an important factor in determining which mode of transport (convection or diffusion) dominates within the IVD and modelling studies have suggested that pro-inflammatory cytokines are of sufficient size (TNFα ∼17.5kDa, IL-1β ∼17.3kDa) that they may be enhanced by convective fluid flow [20], yet experimentally it is less clear which mode dominates the intradiscal transport of pro-inflammatory cytokines. A recent study found that exogenously added pro-inflammatory cytokines were able to penetrate intact rat IVDs when cultured under free swelling (diffusion) conditions [22, 23]. However, another study found that diffusion alone was insufficient to transport exogenously-added dextran (MW: 3kDa), which was an order of magnitude smaller than TNFα, into the nucleus pulposus of ovine caudal IVDs [24]. Together, this suggests that both solute and IVD size are important factors in accurately modeling transport phenomenon relevant to the human condition.

The persistence of inflammatory mediators within the IVD is dictated by the balance between what is being produced and metabolized within the IVD and what is being transported in/out of the tissue. Therefore, in order to accurately investigate how long an elevated presence of inflammatory cytokines persists within the IVD, a model must incorporate both the native cell population and dynamic physiological loading. We previously demonstrated in a bovine caudal organ culture model that the IVD could not recover from a transient exposure to TNFα under static loading conditions [3], suggesting that inflammatory mediators may have persisted throughout the 21-day experiment. However, the experimental conditions in that model may not have been conducive to recovery since vertebral endplates were removed to promote cell viability and static loading was applied. Given the complex nature of transport and cytokine expression, as well as the well-known pro-anabolic effects that dynamic compression has on gene expression [25–27], it remains unknown how long inflammatory mediators will persist within the IVD.

The aims of this study were to investigate (1) whether exogenous TNFα, simulating inflammation of the surrounding spinal tissues, could penetrate an intact IVD, (2) how long inflammatory mediators would persist within the IVD and (3) does the presence of TNFα influence IVD biomechanics. A large animal, bovine caudal IVD organ culture model was used because its size, composition, and metabolism rates are similar to those of human lumbar IVDs [28, 29] and were cultured with endplates retained in a previously described dynamic loading bioreactor system [30]. This model system provides a unique opportunity to investigate questions regarding transport and the interactions between inflammation, mechanical loading and tissue mechanics within the native IVD environment.

## Methods

### Organ Culture Set-Up & Culture Conditions

Bovine caudal IVDs were harvested retaining superior & inferior vertebral endplates from bovine tails obtained from a local abattoir (Green Village Packing Co., NJ). Following isolation, endplates were cleaned with a wound debridement system (Pulsavac, Zimmer, Warsaw, IN) to remove potential blood clots and rinsed with 70% ethanol and washing solution (3% penicillin/streptomycin and 1.5% fungizone in PBS). All IVDs were cultured at 37°C and 5% CO_2_. For all studies, control culture medium consisted of high glucose DMEM, 10% FBS, 50ug/mL ascorbic acid, 1% penicillin/streptomycin, 0.5% fungizone (Fisher-Scientific, Waltham MA), and 1:500 primocin (Invivogen, San Diego, CA) and all TNFα groups were cultured in control medium + 100ng/mL human recombinant TNFα (Invitrogen PHC3016). All reagents were obtained from Invitrogen (Carlsbad, CA) unless otherwise noted. TNFα was used because it is typically expressed following tissue injury [17], is associated with chronic painful conditions of the spine, and is considered an initiator of a larger pro-inflammatory and catabolic cascade in the IVD [31]. TNFα can also be interpreted as a model pro-inflammatory cytokine since it is similar in size to other pro-inflammatory cytokines known to be important in IVD degeneration, such as IL-1β and IL-6.

#### TNFα Transport Study

**Fig 1:**
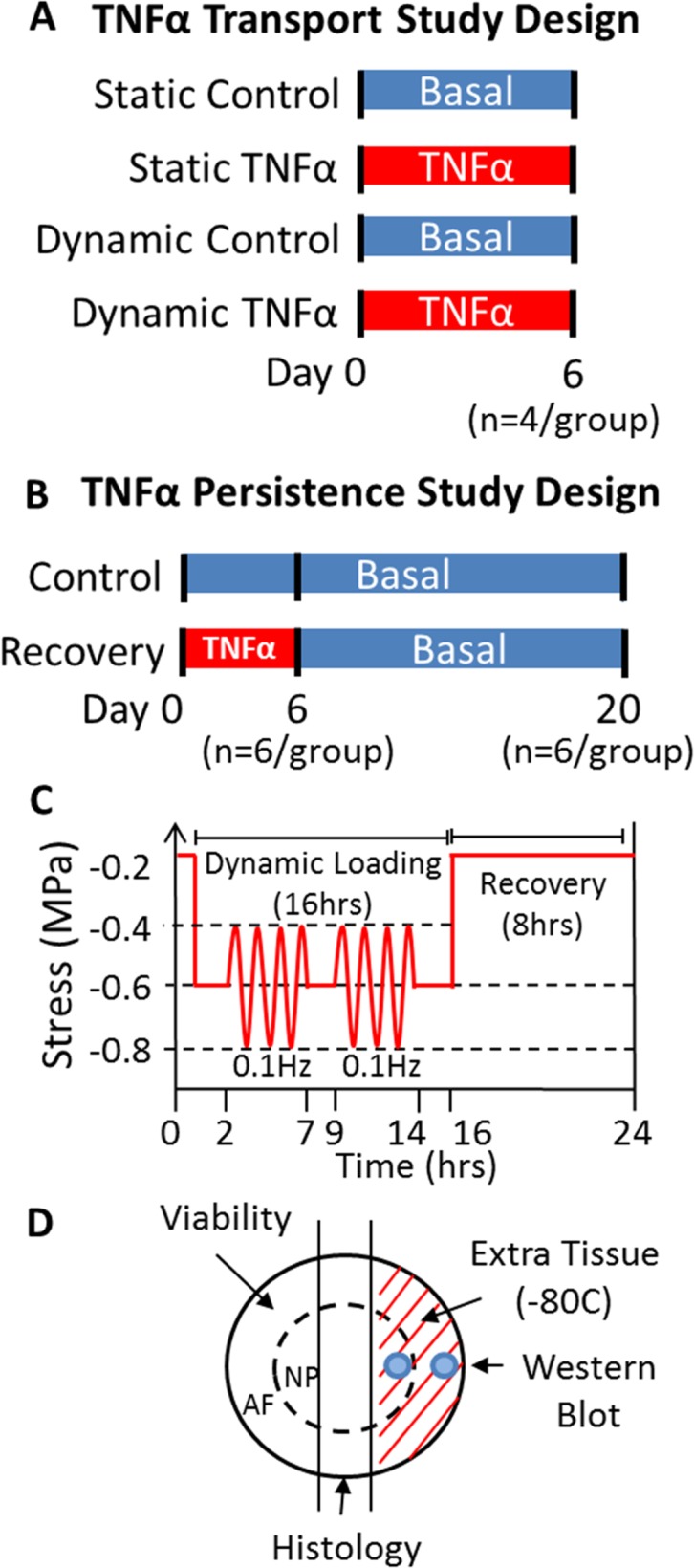
Study Design. (A) TNFα Transport study design to investigate whether TNFα can penetrate an intact IVD and what forms of transport are required. (B) TNFα Persistence study design to investigate how long an inflammatory mediators will remain following removal of TNFα. (C) Simulated physiologic loading conditions used in the TNFα Persistence study. (D) Schematic demonstrating how IVD tissue was used.

This set of experiments investigated whether TNFα could penetrate an intact IVD. All isolated IVDs were assigned to one of four groups consisting of 2 loading conditions (Static/Dynamic) and 2 media conditions (Control Medium/TNFα Medium); Static Control (n = 4), Dynamic Control (n = 4), Static TNFα (n = 4), & Dynamic TNFα (n = 4) (Fig. 1A). Static loading consisted of 24hrs of 0.2MPa static compression and dynamic loading consisted of 8hrs dynamic compression (0–0.8MPa at 0.1Hz) followed by 16hrs static compression (0.2MPa). These loading conditions were chosen to induce either diffusive (static) or diffusive and convective transport (dynamic). Dynamic loading was applied via a previously described organ culture loading system [30]. The culture medium was changed every 3 to 4 days. Dependent variable measurements focused on immunohistochemistry for TNFα.

#### Persistence Study

This set of experiments investigated how long an inflammatory environment persisted following removal of the inflammatory stimulant and whether TNFα influenced IVD biomechanics. Twenty four bovine caudal IVDs were isolated from 6 bovine tails and each IVD was assigned to either Control (Control Medium for 20 days) or Recovery (6 days of TNFα medium followed by 14 days of control medium) groups and cultured for 6 (n = 6/group) or 20 days (n = 6/group) (Fig. 1B). IVD levels were noted and IVDs of different levels were distributed between groups. All IVDs were cultured under simulated physiologic loading applied via a dynamic loading system previously described [30]. The applied simulated physiologic loading consisted of diurnal loading (0.2MPa/0.6MPa) with 2 bouts of ‘exercise’ (0.6±0.2 MPa at 0.1Hz) during the daytime loading (Fig. 1C). The culture medium was changed on day 4, 6, 10, 14, 18. Dependent variable measurements included cell viability at both 6 and 20 days, ELISA for pro-inflammatory cytokine proteins, biomechanical behaviors, histological measurements of structure and composition, and western blot for aggrecan degradation (Fig. 1D).

### Viability

Tissue viability was assessed at 6 and 20 days as previously described [32]. Briefly, tissue was double stained using 3-(4,5-dimethylthiazol-2-yl)-2,5-diphenyltetrazolium bromide (MTT, Sigma-Aldrich, St. Louis, MO), which is metabolized by active mitochondria, and stains viable cells, and 4′,6-diamidino-2-phenylindole (DAPI, Roche Diagnostics, Germany) to stain cell nuclei. Three 10μm thick sections were taken from each sample and photographed at 20x using a microscope. A percent viability (Dual stained cells (DAPI+MTT) / DAPI only cells) was calculated for each image and averaged for each region of each IVD.

### Immunofluorescence & Immunohistochemistry

Immunofluorescence specific for TNFα was performed on IVDs from the Transport study to investigate whether TNFα was able to penetrate the intact IVD. All samples were processed, embedded in plastic and sagittally sectioned (5μm) as previously described [33]. Prior to staining all samples were deplasticized. A primary polyclonal rabbit-anti human TNFα antibody (1:100 ab66579, Abcam Cambrdige, MA) and a goat anti-rabbit Alexafluor 594 secondary antibody (1:700 ab150092, Abcam) were used with omission of primary antibody as a negative control. All slides were counter stained with DAPI. The percentage of positively stained cells was calculated from each 20x image using ImageJ software for each region (annulus fibrosus: AF, 4 images; cartilage end-plates: CEP, 8 images; NP: 8 images).

### ELISA for Pro-Inflammatory Cytokines

Multiple enzyme linked immunosorbet assay’s (ELISA) were used to investigate the amount of human TNFα and bovine pro-inflammatory cytokines (TNFα, IL-1β and IL-6) within the culture medium and how it changed over time. The culture medium from all time points (Days -4, 6, 10, 14, 18 & 20) were analyzed using ELISA’s specific for human TNFα (K15025B-1; Meso Scale Diagnostics, Rockville, MD) and bovine TNFα, IL-1β, IL-6 (N45ZA-1, Meso Scale Diagnostics, Rockville, MD) following manufacturer’s instructions. These specific cytokines were chosen because of their associated with catabolism (TNFα & IL-1β) and pain (IL-6).

### Tissue Mechanics

To characterize whether TNFα induced any changes in IVD biomechanics three mechanical parameters were assessed as previously described [30]. Briefly, the parameters assessed were the total amount of (1) creep and (2) recovery that occurred during each daytime and nighttime period, respectively, and (3) a daily measurement of dynamic stiffness of each IVD, which was calculated from the penultimate cycle of the 2^nd^ dynamic loading period.

### IVD Structure and Composition

To evaluate whether TNFα induced any changes to IVD structure and matrix integrity multiple measurements were assessed and included changes in IVD dimensions, histology and aggrecan degradation via western blot. IVD height and diameter were recorded at set-up and takedown of the experiment. Height changes were monitored throughout culture and the total height change was normalized to the equilibrium height (the IVD height after the IVD has undergone one complete diurnal cycle and fully recovered) as previously described [30]. To assess how TNFα influenced IVD composition, sagittal sections (5μm) from each group (Control & Recovery) at each timepoint (6 & 20 Days) were stained with picosirius red / alcian blue. A western blot was done using an antibody specific to the G1 region of aggrecan as previously described [34], briefly the amount of GAG in each tissue extract was quantified and an equal weight of GAG (1.5ug) from each sample was loaded into each lane.

### Statistics

#### Viability & TNFα Immunohistochemistry

A one-way ANOVA was used to compare the percent viability between time points within each region, and the percentage of TNFα positive cells between groups in the Transport study. An un-paired t-test was used to compare the amount of pro-inflammatory cytokines at each time point between control and recovery groups. *Tissue mechanics*: Since for the first six days of culture there were no differences between the recovery and the 6-day TNFα groups, the two values for each animal were averaged and the averaged values for the control and TNFα groups were compared with a paired t-test. An un-paired t-test was used to compare all mechanical parameters after day 6 between control and recovery groups. All statistical analysis were conducted with GraphPad Prism 3 (La, Jolla, CA) with p<0.05 considered significant (*) and p<0.08 considered a trend (ⱡ).

## Results

### Immunofluorescence & Immunohistochemistry

#### Transport Study / TNFα Immunofluorescence

**Fig 2:**
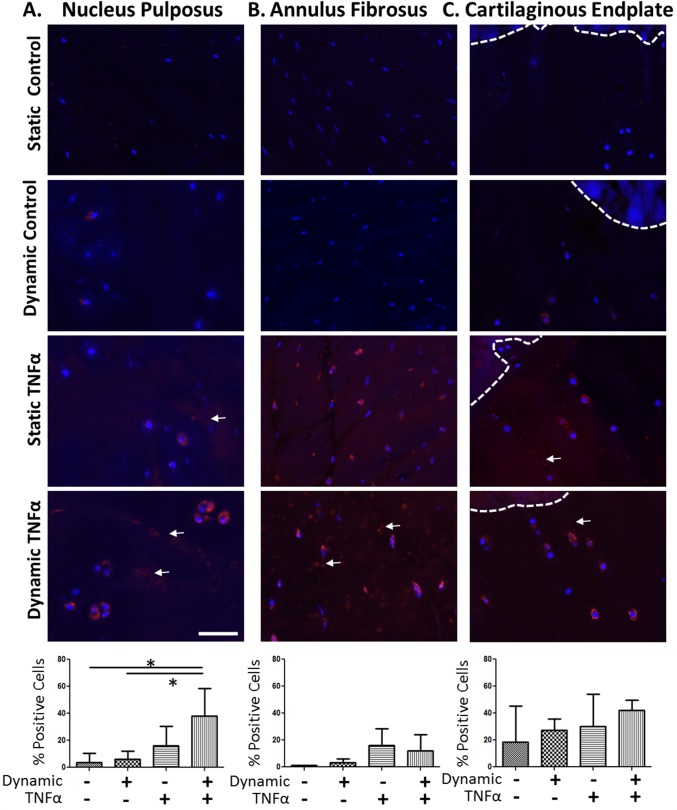
TNFα Transport. Representative 20x images and quantification of the % TNFα positive stained cells in the **(A)** Nucleus Pulposus, **(B)** Cartilaginous Endplate (dashed line demarcates the boney endplate), and **(C)** Annulus Fibrosus. Results demonstrate that dynamic loading (convective transport) is required for TNFα to penetrate into the NP of intact IVDs (i.e., uninjured endplate/IVD/endplate sections) and suggests transport likely occurred via the CEP. Omission of primary antibody was used as a negative control. * = p<0.05.

Dynamic loading and the associated convective transport greatly enhanced the amount of TNFα present within intact IVDs. The only significant differences were observed within the NP region, as it is the most transport limited region of the IVD. In the NP, the Dynamic TNFα group (38.1±20.3%) had the greatest %TNFα positive cells which was significantly greater than both the Static (3.8±6.6%) and Dynamic Controls (6.0±6.3%) and non-significantly greater than the Static TNFα group (16.3±14.3%) (Fig. 2). In the CEP, there were no significant differences in the %TNFα cells between groups with Static Control, Dynamic Control, Static TNFα, and Dynamic TNFα expressing 18.5±27%, 27.3±8.3%, 29.9±24.1% & 42.0±7.5%, respectively. In the AF, there were also no significant differences in percent TNFα positive cells with Static Control, Dynamic Control, Static TNFα, & Dynamic TNFα expressing 1.1±.25%, 3.4±3.3%, 16.1±14.3% & 12.2±11.9%, respectively. In all regions of the TNFα treated IVDs there was diffuse TNFα staining within the matrix (Fig. 2, white arrows), however this was not quantified. The significant increase in percentage of TNFα positive cells in the Dynamic TNFα group demonstrates that exogenous TNFα was able to penetrate the intact IVD and implies that dynamic loading and the associated convective transport are required for TNFα to penetrate an intact large animal NP.

### Viability

**Fig 3:**
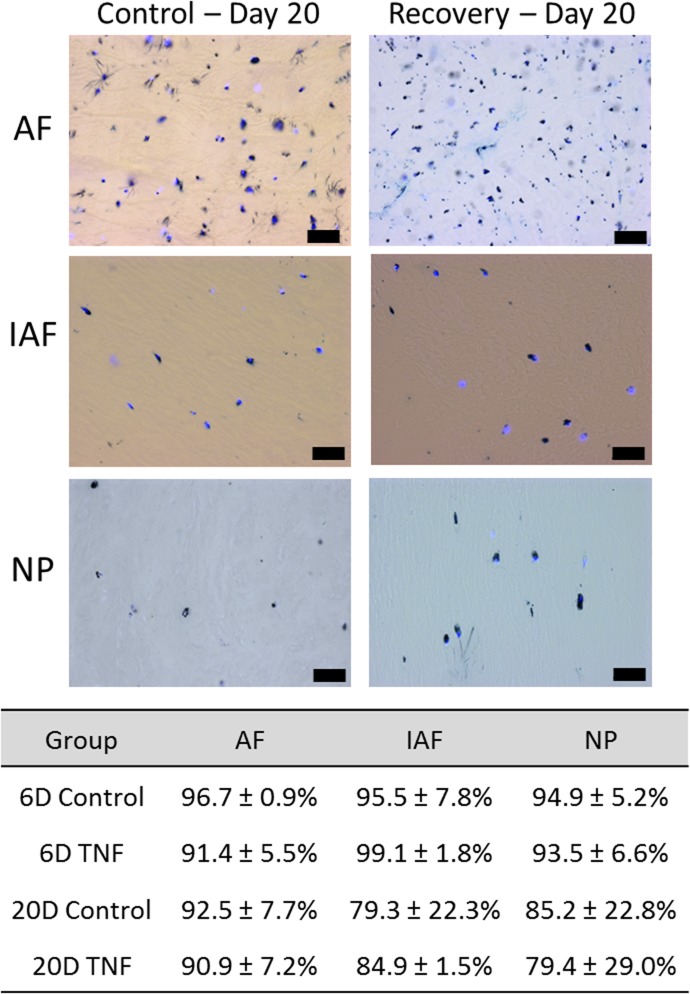
Tissue Viability. Representative viability images and quantification of IVD viability for both Control and Recovery groups after 20 days of culture in the different regions of the IVD; Annulus Fibrosus (AF), Inner Annulus Fibrosus (IAF) Nucleus Pulposus (NP) Scale bar = 50μm.

All IVDs remained viable at both 6 and 20 days with all regions maintaining at least 79% viability in all regions (Fig. 3).

### ELISA

**Fig 4:**
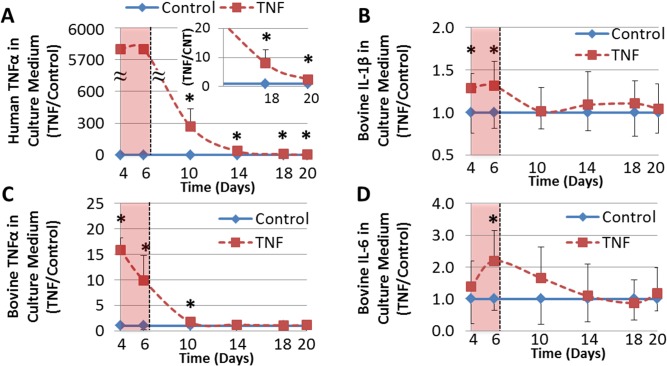
Cytokines in Culture Media. Changes in the amount of human and bovine pro-inflammatory cytokines present within the culture medium over time, normalized to control (**A**) Human TNFα (**B)** Bovine IL-1β (**C)** Bovine TNFα (**D)** Bovine IL-6. Shaded Pink regions indicate time while exogenous human TNFα was present in culture medium (TNFα was removed on day 6). Results demonstrate a sustained release of human TNFα into the culture media and a transient response of the bovine cytokines, returning to baseline by day 14.

The presence of human TNFα induced an increase in all the measured bovine pro-inflammatory cytokines (TNFα, IL-1β & IL-6) compared to time-matched controls (Fig. 4). Bovine IL-6 and TNFα remained elevated within the culture medium at day 10 and returned to control levels by day 14 and IL-1β returned to control levels by day 10. Human TNFα remained significantly elevated in the culture medium throughout the entire culture duration compared to time-matched controls.

### Tissue Mechanics

**Fig 5:**
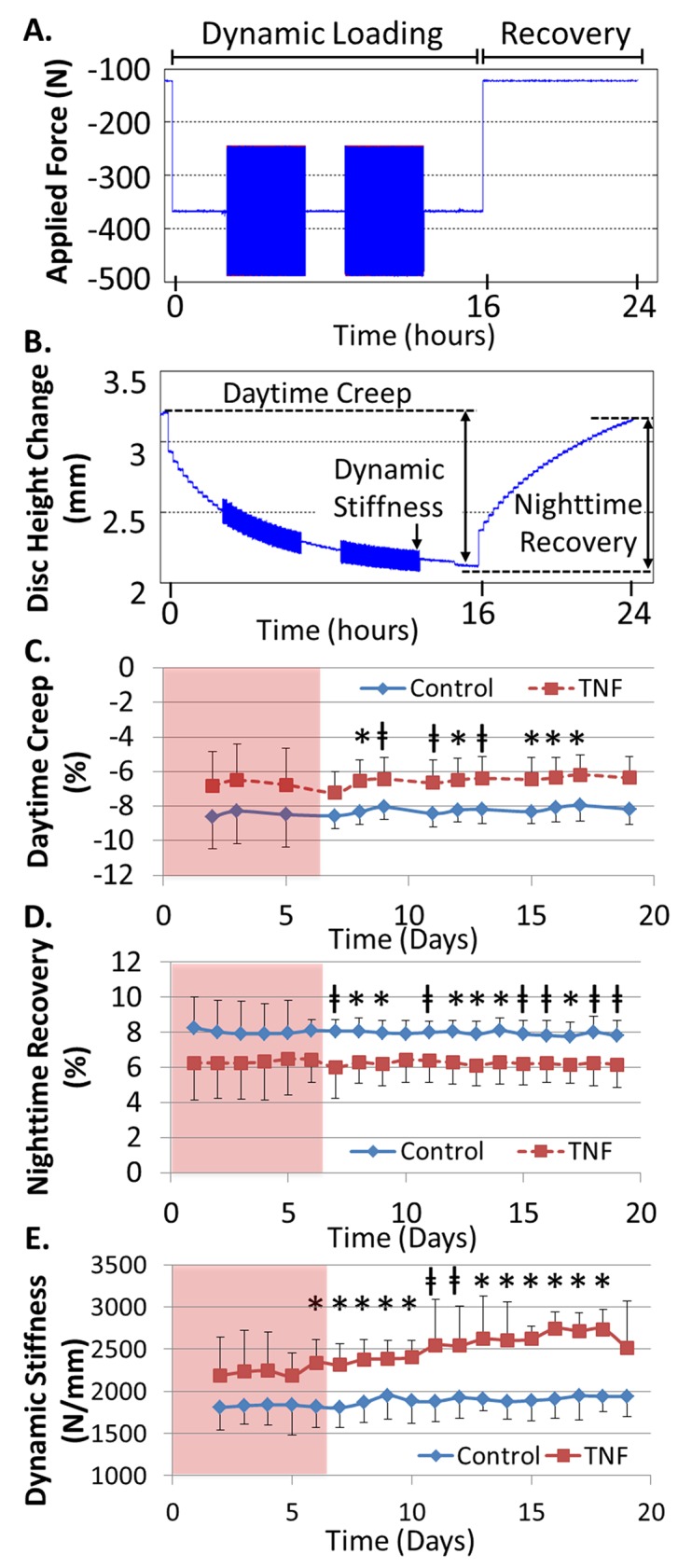
TNFα influences IVD Biomechanics. An example of the (**A**) applied loading and (**B**) resulting displacements of cultured IVDs. Three mechanical parameters were assessed during each 24hr period. The percent change in height during the (**C**) daytime creep and (**D**) nighttime recovery over the culture period. (**E**) Changes in dynamic stiffness throughout the culture period. The pink shaded region indicates time while exogenous TNFα was present in culture medium (TNFα was removed on day 6). * = p<0.05 & ⱡ = p<0.08.

Biomechanically, TNFα induced changes representative of tissues stiffening in all assessed mechanical parameters (reduced diurnal displacements and increased dynamic stiffness, Fig. 5A&B). The Recovery group had a reduction in both the total amount of daytime creep and night time recovery, which was significant or had a trend after day 6 compared to time-matched controls (Fig. 5C&D). The Recovery group also demonstrated an increased dynamic stiffness starting at day 6 (Recovery: 2336.4±277.1 N/mm, Control: 1813.8±239.1 N/mm) which continued to stiffen throughout the culture period compared to time-matched controls (Fig. 5E). There was no significant change in the dynamic stiffness of the Control group throughout the culture period, demonstrating that the applied loading was able to maintain IVD mechanics.

### IVD Structure and Composition

**Fig 6:**
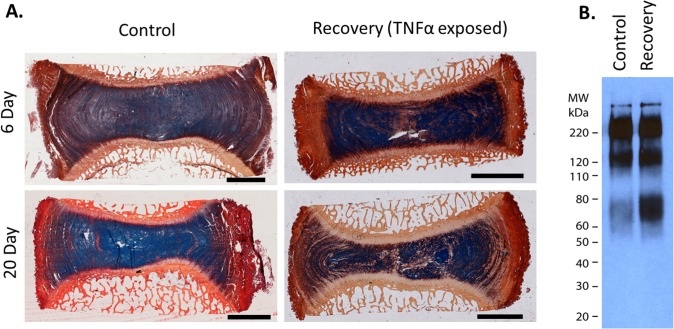
TNFα induces Aggrecan Breakdown. **(A)** Picrosirius red & Alcian blue staining of Control and Recovery groups. Recovery involved 6 days of TNFα exposure followed by 14 days with control media. Recovery group exhibited increased picrosirius red staining and a loss of Alcian blue staining intensity in the nucleus pulposus, suggestive of aggrecan loss. (**B)** Western blot for the aggrecan G1 domain demonstrating an increase in aggrecan matrix degradation products at day 20. Scale bar = 5mm

There were no significant differences in disc height loss (<10% for all groups) between Control and Recovery groups at either time point; Control Day 6: −8.7±2.9%, Recovery Day 6: −8.5±6.4%, Control Day 20: −9.5±5.8%, Recovery Day 20: −8.1±4.3%, suggesting that tissue compaction was not responsible for increased IVD stiffness in the Recovery group. Picosirius Red/Alcian Blue staining showed a more fibrous structure within the NP (increased collagen staining) and reduced alcian blue staining suggestive of increased aggrecan degradation (Fig. 6A). Western blot confirmed that there was an increase in the amount of aggrecan breakdown products within the NP region of TNFα treated IVDs (Fig. 6B).

## Discussion

This study developed an inflammatory IVD degeneration model to investigate whether TNFα can penetrate an intact IVD, how long inflammatory mediators will persist and whether the short-term presence of TNFα can significantly alter disc mechanics. Results demonstrated that dynamic loading, and the associated convective transport, significantly enhanced the penetration of TNFα into intact bovine IVDs (endplate/IVD/endplate) which induced transient increases in the production of all bovine pro-inflammatory cytokines measured (TNFα, IL-1β and IL-6). TNFα treatment also increased aggrecan degradation which likely contributed to the permanent alterations in the biomechanical behavior of IVDs, which was representative of tissue stiffening (progressive increase in dynamic stiffness). Overall this model demonstrated that a transient TNFα challenge can cause lasting biomechanical changes independent of a persistent presence of inflammatory mediators.

Exogenous TNFα was able to penetrate an intact IVD and this was clearly visualized with immunohistochemistry staining for TNFα. Similar amounts of positive TNFα staining were observed in the AF region of both the Static TNFα (16.1±14.3%) and Dynamic TNFα groups (12.2±11.9%), suggesting that a similar amount TNFα entered via the AF. However, in the endplate region the Dynamic TNFα group (42.0±7.6%) had a greater amount of TNFα staining than the Static TNFα group (29.0±24%) suggesting that a majority of the TNFα present in the NP occurred through the CEP of the IVD and not via the AF. This suggestion of preferential transport occurring via the endplate contrasts a recent study which assessed the hydraulic permeability of different IVD tissues and found that the AF has an ∼10 fold greater permeability than the CEP [35]. However, other factors are also important when assessing the cumulative amount of TNFα transport such as the shorter transport distances between the CEP & NP as well as the larger exposed surface area of the CEP compared to the AF. The addition of 8hrs of cyclic loading approximately doubled the amounts of TNFα found in NP regions. We speculate that the diurnal changes in IVD height (which are relatively large), and the corresponding bulk fluid flux likely had the greatest contribution to the convective transport of TNFα since the amount of height change (and fluid flux) occurring during the cyclic loading periods were relatively small in comparison. However, it is possible that the relatively short application of dynamic loading directly increased the amount of TNFα transported into the NP as dynamic loading of a porous solid matrix can effectively ‘pump’ solutes into the porous matrix giving rise to concentrations which greatly exceed those attainable under passive diffusion alone [36]. Some positive TNFα staining was observed in both the static and dynamic controls where exogenous human TNFα was not added, suggesting that the anti-human TNFα antibody used cross-reacted with bovine TNFα. However, the levels of positive TNFα staining were very low in both controls and would suggest that the applied loading conditions did not induce significant amounts of TNFα expression and therefore the addition of dynamic loading, and the associated convective transport, is responsible for the increased amount of TNFα positive cells in the NP region of the Dynamic TNFα group.

The bovine pro-inflammatory cytokines TNFα, IL-1β, IL-6 that were present in the culture medium returned to baseline levels within 4–8 days following TNFα removal. While human TNFα remained significantly elevated compared to control throughout the entire culture duration (14 days after TNFα removal) the observation that it continued to decrease over time suggests it would also eventually return to baseline. The slow outward transport of TNFα is likely influenced by two factors. First, the high initial dose of TNFα (100ng/mL) will likely cause it to persist for longer duration. This dose is considered hyper-physiologic for other tissues; however, this dose has been used and justified in previous tissue culture models [3, 22, 37, 38] and the concentrations that occur in IVD degeneration are unknown. Second, TNFα may have been sequestered within the matrix, especially as molecular charge is known to significantly influence the transport of molecules within the IVD and cartilage [39]. The low pH within the degenerated NP (6.8-6.2pH) [40] is below the theoretical isoelectric point (the pH at which a protein has no charge) of multiple pro-inflammatory cytokines (TNFα: pI∼7, IL-8: pI∼9) which would create an electrostatic attraction between the positively charged cytokines and the negatively charged proteoglycans within the IVD, slowing the outward transport. The transience of the bovine inflammatory mediators suggests that the autocrine disc-cell mediated inflammatory response may have a limited ability to maintain elevated levels of pro-inflammatory cytokines, and highlights a possible role for macrophages or other immune cells to serve as a source of pro-inflammatory cytokines. However, this model only observed the acute response to non-pathologic loading in a relatively healthy IVD, and it is possible that production of pro-inflammatory cytokines by the native IVD cells is increased in degeneration due to the accumulation of structural defects and the resulting alteration in mechanical behavior.

TNFα is known to dramatically enhance catabolic processes in the IVD, however it was unknown if and/or how quickly elevated catabolism would influence IVD mechanics. TNFα induced biomechanical changes representative of tissue stiffening (reduced diurnal displacements and increased dynamic stiffness). The progressive change in Dynamic Stiffness, which was the most notable biomechanical change, was associated with the accumulation of matrix degradation and the delay before measureable change were observed at day 6 is consistent with sufficient time being required for matrix degradation to accumulate. At both 6 and 20 days, TNFα treated IVDs had increased collagen staining within the NP, and a reduced alcian blue staining intensity indicative of aggrecan loss. Western blot confirmed that TNFα treated IVDs had increased amounts of aggrecan degradation after 20 days of culture. Interestingly, there were no differences in the height loss between all groups (<10%), suggesting that the increase in IVD stiffness was not associated with tissue compaction. This increase of aggrecan degradation together with no difference in height loss between groups suggests that the increased stiffness of TNFα treated IVDs may be a result of altered load carriage by shifting more load to the AF due to a loss of NP pressurization. This concept of altered load carriage is consistent with the altered stress distribution observed in advanced degeneration [41]. While the reduced diurnal displacements are consistent with an increased dynamic stiffness we cannot rule out the influence of biologic variation of the selected IVDs in contributing to the differences in diurnal displacements as there was a consistent, although non-significant, shift between TNFα and Control IVDs throughout the culture period. It is also difficult to directly translate the relative speed through which TNFα influenced disc mechanics to the human condition as the dose used here was hyper-physiologic. However, these results suggest that the mechanical behavior of the IVD is altered relatively quickly following aggrecan degradation and support the hypothesis that exogenous pro-inflammatory cytokines can penetrate an intact IVD and contribute to the initial weakening of the IVD structure.

IVD degeneration has been described as “frustrated healing”, where structural damage accumulates as the IVD experiences high loads. The weakened structure then alters the load distribution and leads to an abnormal (catabolic) metabolism which further weakens the matrix [41, 42]. Structural disruption was postulated to be “the essential non-reversible step” that transitions normal ageing into this ‘frustrated healing’ process that accelerates IVD degeneration [42]. Our results support this conceptual model and suggest that a transient exposure to elevated levels of pro-inflammatory cytokines, as might occur from injury and/or inflammation of the surrounding spinal tissues, can lead to matrix breakdown which can directly and rapidly alter the mechanical behavior of the IVD. We therefore propose that exposure to pro-inflammatory cytokines, however brief, may be a mechanism initiating degenerative changes through weakening the matrix and beginning the transition from normal ageing to the ‘frustrated healing’ process and accelerated degeneration.

In conclusion, this study used exogenous TNFα as a model cytokine to investigate how the interactions between mechanical loading and inflammation can contribute to the progression of IVD degeneration. We demonstrated (1) that exogenous TNFα can penetrate an intact IVD, (2) if the healthy IVD becomes infiltrated by inflammatory mediators they persist but can return to baseline levels within 4–8 days if the source is removed, and (3) that pro-inflammatory cytokines can rapidly alter IVD mechanics. These substantial and persistent matrix changes suggest that pro-inflammatory cytokine exposure may be a mechanism initiating degenerative changes and not only a consequence of injury and disease. This inflammatory model of IVD degeneration showed progressive matrix and mechanical changes and may be useful for evaluating future therapies.
